# Regulation of cancer cell adhesion, progression, and epithelial-to-mesenchymal transition by N-glycosylation

**DOI:** 10.1093/pcmedi/pbag002

**Published:** 2026-01-16

**Authors:** Jianguo Gu

**Affiliations:** Division of Regulatory Glycobiology, Graduate School of Pharmaceutical Sciences, Tohoku Medical and Pharmaceutical University, Sendai 981-8558, Japan; Institute of Molecular Biomembrane and Glycobiology, Tohoku Medical and Pharmaceutical University, Sendai 981-8558, Japan

Cancer progression is a highly complex process, and cellular adhesion plays a crucial role in enabling cancer cells to grow, invade, and metastasize. This process is regulated not only by adhesion molecules but also by post-translational modifications, with N-glycosylation being particularly important. N-Glycosylation significantly affects multiple stages of cancer development, including cell adhesion, epithelial-to-mesenchymal transition (EMT), and cancer cell growth and spread. This commentary briefly discusses the role of N-glycosylation in regulating cancer cell adhesion, progression, and EMT, and how targeting this process could provide new insights into precision medicine therapeutic strategies.

## N-Glycosylation and its role in cellular functions

**Figure 1:**
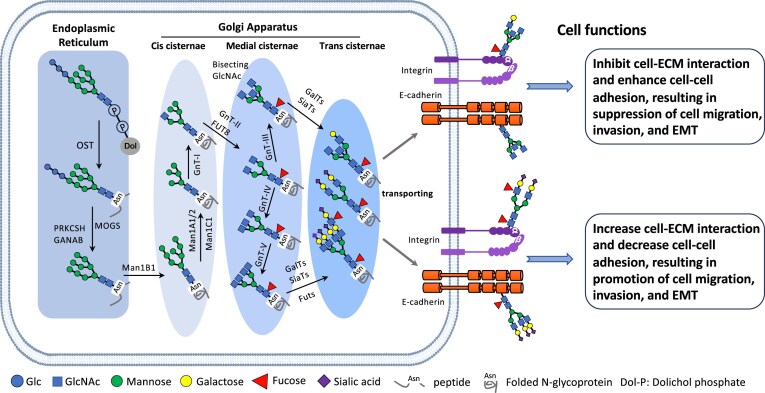
Biosynthetic pathway for N-glycans and their regulatory cell functions. The initiation of N-glycosylation is the process of attaching an oligosaccharide chain (Glc3Man9GlcNAc2) linked with dolichol phosphate in the endoplasmic reticulum (ER) to an Asn residue within a specific sequence (Asn-X-Ser/Thr, where X is any amino acid except proline) by the oligosaccharyltransferase (OST) complex in mammals. These folded N-glycoproteins are then processed in the Golgi apparatus, where additional glycan remodeling occurs through the actions of glycosidases and glycosyltransferases. For example, the addition of a bisecting GlcNAc catalyzed by N-acetylglucosaminyltransferase III (GnT-III) prevents further processing of N-glycans, thereby blocking the synthesis of GlcNAc-branched N-glycans by GnT-IV and GnT-V, which sequentially influence terminal modifications such as fucosylation and sialylation to affect cell functions. MOGS: Mannosyl-oligosaccharide glucosidase; PRKCSH: protein kinase C substrate 80K-H; GANAB: glucosidase II alpha subunit; Man: alpha-mannosidase; Fut: fucosyltransferase; GnT: *N*-acetylglucosaminyltransferase; GalT: galactosyltransferase; SiaT: sialyltransferase.

N-Glycosylation is the process of attaching an oligosaccharide chain (Glc3Man9GlcNAc2) to the nitrogen atom of an asparagine (Asn) residue within a specific sequence (Asn-X-Ser/Thr, where X is any amino acid except proline) by the oligosaccharyltransferase complex in mammals. This modification occurs in the endoplasmic reticulum and is then processed in the Golgi apparatus, where additional glycan remodeling takes place through the actions of glycosidases and glycosyltransferases (Fig. 1). Ultimately, glycoproteins with mature N-glycan structures, such as those involved in fucosylation and sialylation, are transported to the cell membrane or to the extracellular space. N-Glycans help regulate protein folding, stability, function, and trafficking, and they also play roles in cell signaling, immune response, and cell-to-cell communication.

It is estimated that there are >2 000 N-glycan structures [1], resulting from various combinations of primary monosaccharide species, anomeric and linkage variations, branching or extensions, and other modifications like sulfate or phosphate additions. Over the past decade, thousands of N-glycoproteins have been identified by detecting their glycosite-containing peptides using mass spectrometry. It has been reported that >30 000 unique human glycosite-containing peptides have been identified by mass spectrometry, representing >14 000 unique N-glycosites from >7200 N-glycoproteins [2]. Therefore, mapping tissue N-glycans and decoding tissue-specific protein N-glycosylation can reveal increasingly informative changes in diseases and the biological processes behind them. Nevertheless, the study of glycoproteomics is important but is fraught with challenges due to the low abundance and intricate structures of glycosylation [3, 4].

In cancer, alterations in N-glycosylation patterns are common, and changes in glycan structures can affect various cellular behaviors [5]. This includes modified cell adhesion, migration, and invasion, all of which promote cancer progression. Identifying distinct, highly abundant N-glycans in different tissue types that are significantly altered in cancer may reveal disease mechanisms or even serve as early markers of tumor aggressiveness.

## N-Glycosylation and cancer-cell adhesion

Cell adhesion is crucial for tissue structure and cell interactions. In healthy tissues, molecules such as integrins, cadherins, and selectins help maintain homeostasis by regulating cell–cell and cell–matrix adhesions. However, in cancer, these adhesion processes are often impaired, enabling tumor cells to detach from the primary site and spread to other organs.

N-Glycosylation plays an important role in regulating the function of cell-adhesion molecules [6, 7]. A well-studied example is the adhesion molecule E-cadherin, which is essential for maintaining epithelial structure. E-Cadherin’s adhesive ability is significantly influenced by its N-glycosylation patterns. Changes in glycosylation—such as exposing terminal sialic acids or adding fucose—can lead to a loss of adhesive function, disrupting the epithelial monolayer and causing cellular dissociation (Fig. 1), a hallmark of EMT. Additionally, N-glycosylation also affects integrin function. Integrins are cell-surface receptors that mediate interactions between cells and the extracellular matrix (ECM). Abnormal N-glycosylation of integrins has been associated with increased cell migration and invasion, which are key steps in cancer metastasis [8]. For example, in breast cancer, the N-glycosylation pattern of integrin β1 is altered, thereby strengthening interactions with ECM components such as fibronectin and collagen. These modifications support the invasive behavior of cancer cells by enhancing integrin-mediated signaling pathways that promote cell migration. Similarly, in colorectal cancer, changes in the glycosylation of selectins influence their ability to mediate leukocyte adhesion and recruitment, contributing to tumor inflammation and metastasis [7].

## N-Glycosylation in EMT and cancer progression

EMT is a biological process in which epithelial cells lose their polarity and adhesion to neighboring cells, gaining a mesenchymal phenotype that is more migratory and invasive [9]. EMT is a crucial step in cancer metastasis, allowing cancer cells to detach from the primary tumor, invade surrounding tissues, and migrate to distant organs. N-Glycosylation plays a key role in regulating EMT. For example, the glycosylation status of key EMT regulators, such as E-cadherin and β-catenin, determines whether epithelial integrity is maintained or lost. A decrease in E-cadherin and an increase in mesenchymal markers such as vimentin and fibronectin are common indicators of EMT, and these changes often involve different glycosylation patterns.

One of the key glycosylation modifications that support EMT is sialylation [4]. Adding sialic acid to glycan structures can influence cell–cell and cell–ECM interactions, thereby enhancing the migratory and invasive abilities of cancer cells. The β-galactoside α2,6 sialyltransferase 1 (ST6GAL1), an enzyme that primarily forms terminal α2,6 sialic acid linkages on N-glycans (Fig. 1), is specifically upregulated during transforming growth factor-β (TGF-β)-induced EMT. Knockdown of ST6GAL1 clearly inhibits EMT, accompanied by a corresponding increase in E-cadherin. Fucosylation, another form of glycosylation, also plays a significant role in EMT. Additionally, core fucosylation of integrins, catalyzed by fucosyltransferase 8 (Fig. 1), can increase their affinity for ECM components, thereby promoting cell migration and invasion.

A key example of N-glycosylation’s role in cancer progression is the modification of growth factor receptors, such as the epidermal growth factor receptor (EGFR) [10]. EGFR plays a crucial role in tumor development by transmitting signals that promote cell growth, survival, and mobility. N-Glycosylation of EGFR can influence its ligand-binding ability, internalization, and activation. In lung cancer, abnormal N-glycosylation of EGFR has been linked to increased receptor signaling and enhanced tumorigenic traits.

## Therapeutic implications and future directions

Since N-glycosylation plays a vital role in cancer cell adhesion, progression, and EMT, it opens new avenues for treatment. Targeting enzymes that add or modify glycans—such as glycosyltransferases and glycosidases—provides a promising approach to prevent cancer spread. For instance, inhibitors of sialyltransferases have shown promise in reducing the migratory and invasive abilities of cancer cells in preclinical studies [11]. Additionally, the development of glycan-targeting antibodies is an expanding area of research. These antibodies can be engineered to specifically recognize and bind to altered glycan structures on tumor cells, thereby blocking their interactions with adhesion molecules and ECM components [12]. Furthermore, the Bertozzi group has developed a novel antibody–enzyme therapeutic, a bisialidase fusion protein (E-602), in which trastuzumab (an anti-HER2 monoclonal antibody) is conjugated to a neuraminidase that cleaves sialic acids on HER2-positive cancer cells. This local desialylation at the tumor site results in immune cell activation, as demonstrated in preclinical studies [13]. It is currently being tested in a Phase I/II trial (NCT05259696) for dose escalation and dose expansion. This could potentially decrease tumor cell invasiveness and improve immune system detection of cancer cells. Understanding the molecular links between N-glycosylation and cancer progression may provide valuable insights into potential therapeutic targets. In addition, glycosphingolipids are also targets. GD2 is an intermediate member of the acidic glycosphingolipid family and is overexpressed in melanoma, glioma, neuroblastoma, and small cell lung cancer. Dinutuximab, a chimeric human anti-GD2 mAb, was approved by the Food and Drug Administration for the treatment of patients with high-risk neuroblastoma in 2015 (NCT00026312) [14].

In cancers, altered glycosylation structures, known as tumor-associated carbohydrate antigens (TACAs), are cancer-type-specific and are expressed on different cell-surface molecules. Thus, TACAs are potential tumor glyco-biomarkers, glycoimmune checkpoints, and therapeutics [15]. Although clinical trials testing these therapies are still in the early stages, they show promise for future cancer treatments, particularly in preventing metastasis and improving patient outcomes.
